# Multimodal striatal neuromarkers in distinguishing parkinsonian variant of multiple system atrophy from idiopathic Parkinson's disease

**DOI:** 10.1111/cns.13959

**Published:** 2022-09-01

**Authors:** Huize Pang, Ziyang Yu, Hongmei Yu, Miao Chang, Jibin Cao, Yingmei Li, Miaoran Guo, Yu Liu, Kaiqiang Cao, Guoguang Fan

**Affiliations:** ^1^ Department of Radiology The first Affiliated Hospital of China Medical University Shenyang China; ^2^ School of Medicine Xiamen University Xiamen China; ^3^ Department of Neurology The first Affiliated Hospital of China Medical University Shenyang China

**Keywords:** Idiopathic Parkinson's disease, machine learning, multimodal MRI, parkinsonian variant of multiple system atrophy, striatum

## Abstract

**Aims:**

To develop an automatic method of classification for parkinsonian variant of multiple system atrophy (MSA‐P) and Idiopathic Parkinson's disease (IPD) in early to moderately advanced stages based on multimodal striatal alterations and identify the striatal neuromarkers for distinction.

**Methods:**

77 IPD and 75 MSA‐P patients underwent 3.0 T multimodal MRI comprising susceptibility‐weighted imaging, resting‐state functional magnetic resonance imaging, T1‐weighted imaging, and diffusion tensor imaging. Iron‐radiomic features, volumes, functional and diffusion scalars of bilateral 10 striatal subregions were calculated and provided to the support vector machine for classification

**Results:**

A combination of iron‐radiomic features, function, diffusion, and volumetric measures optimally distinguished IPD and MSA‐P in the testing dataset (accuracy 0.911 and area under the receiver operating characteristic curves [AUC] 0.927). The diagnostic performance further improved when incorporating clinical variables into the multimodal model (accuracy 0.934 and AUC 0.953). The most crucial factor for classification was the functional activity of the left dorsolateral putamen.

**Conclusion:**

The machine learning algorithm applied to multimodal striatal dysfunction depicted dorsal striatum and supervening prefrontal lobe and cerebellar dysfunction through the frontostriatal and cerebello‐striatal connections and facilitated accurate classification between IPD and MSA‐P. The dorsolateral putamen was the most valuable neuromarker for the classification.

## Graphical abstract

The machine learning algorithm applied to multimodal striatal dysfunction depicted dorsal striatum and supervening prefrontal lobe and cerebellar dysfunction through the frontostriatal and cerebello‐striatal connections and facilitated accurate classification between IPD and MSA‐P at the individual level. The dorsolateral putamen was the most valuable neuromarker for the classification.

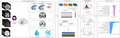

## 1 INTRODUCTION

Idiopathic Parkinson's disease (IPD) and multiple system atrophy (MSA), especially the parkinsonian variant (MSA‐P), are two neurodegenerative diseases that manifest as clinical symptoms but pose difficulty in the differential diagnosis. Unlike IPD, MSA‐P patients have faster progression, poorer prognosis, lesser sensitivity to dopamine treatment, and unsuitability for deep brain stimulation.^1^ Considering the prognosis and therapeutic differences between the two phenomena, an accurate separation in the early to moderately advanced stage is clinically essential.

Currently, the clinical diagnosis of IPD and MSA‐P is based on the history and neurological examinations, allowing misdiagnosis due to individual differences and subjective factors.^2^ Magnetic resonance imaging (MRI) is the most applied non‐invasive examination for diagnosis and a series of specific diagnostic signs, such as lack of “swallow‐tail” sign for IPD diagnosis and “putaminal rim” sign for MSA diagnosis, have been proposed.^3^, ^4^ However, the value of these signs in clinical diagnosis is inconsistent, partially due to their existence in normal aging people and non‐detectability in a subgroup of IPD and MSA‐P patients due to the short period of the disease.^5^, ^6^ As a result, finding sensitive and early neuromarkers that may improve the accuracy of the clinical diagnostic criteria is imperative. From a neuropathological perspective, the central role of striatum underlying the pathophysiology of both IPD and MSA has been verified.^7^ In addition, PD patients exhibited varied dopamine transporter loss and iron deposition patterns in the striatum of MSA patients on positron emission tomography (PET) and iron‐sensitive MRI, which was ascribed as striatum's role in differential diagnosis.^6^, ^8^, ^9^

Therefore, striatal dysfunction in IPD and MSA has been studied.^10^, ^11^, ^12^, ^13^, ^14^ Aberrant functional activity and connectivity were observed on resting‐state functional MRI (rs‐fMRI),^10^, ^13^ brain atrophy was found on T1‐weighted images (T1WIs),^11^, ^12^, ^13^ and microstructure changes were visible on diffusion‐weighted images (DWI).^12^, ^14^ Nevertheless, these findings were group‐level, causing restriction to clinical usage. The development of a machine learning method for the interpretation of imaging biomarkers provides a possibility for individualized prediction.^15^ Most studies have included a single modality of MRI to prediction. For example, Baggio et al. discriminated MSA from IPD based on rs‐fMRI and obtained an accuracy of 77.17%.^16^ Other researchers established discriminative models of PD and MSA using DWI sequence and obtained an accuracy of 78%.^17^ One of our previous works reported discriminative model based on SWI, yielding AUC from 0.583 to 0.788.^6^ The moderate performance was partially due to the limited information obtained through a single modality. While, other studies reported preferable performance based on a single modality MRI. For example, researchers established decision tree using T1WI and DWI sequence to differentiate PD and MSA.^18^, ^19^, ^20^ In addition, other researchers applied logistic model to advanced DWI and obtained excellent diagnostic performance.^21^ However, it may lead to biased model performance estimates with a small sample size in the above studies. Compared with single modality sequence, multimodal MRI increases the accuracy to collect the disease‐relevant tissue measurements and determine the relative predictive strengths of disease‐related functional and structural alterations.^22^ Nemmi et al.^22^ utilized multimodal MRI data to establish a discrimination model of PD and MSA but failed to separate the MSA‐P subtype. It is rather challenging to differentiate MSA, especially MSA‐P subtype, as opposed to cerebellar subtype (MSA‐C), from IPD as both exhibit parkinsonian symptoms. Studies on differential diagnosis between MSA‐P and IPD are sparse. Researchers applied discriminant analysis to a combination of T1WI, T2*WI, and DWI and achieved an accuracy of >95%.^23^, ^24^ However, the reported results were based on validation dataset. According to the guideline on artificial intelligence, an independent testing dataset is required for evaluating final results.^25^ Besides, Tsuda et al.^26^ used neural network based on T1WI and MRS, providing an AUC of 0.775. The moderate diagnostic accuracy may be attributed to the partial information provided by the limited MRI modality. Therefore, in order to transpose an unbiased and high accuracy classification model, a discriminative model using a combination of multimodal MRI information on the training dataset with large sample size and an evaluation model on an independent testing dataset is essential.

In the present study, we aimed to construct an automatic differential diagnosis model based on multimodal striatal alterations for the classification of IPD and MSA‐P patients in early to moderately advanced stage that can be translated into a clinical setting. In addition, we aimed to find striatal neuromarkers by analyzing the feature weights involved in model prediction.

## 2 MATERIALS AND METHODS

### 2.1 Subjects

A total of 77 patients with IPD and 75 patients with MSA‐P were included and diagnosed by advanced movement specialists at the outpatient clinic of the Department of Neurology of the First Affiliated Hospital of China Medical University from February 2017 until April 2020. Inclusion criteria were: (1) a clinical diagnosis of probable MSA or IPD according to consensus criteria.^27^, ^28^ MSA‐P subtypes were diagnosed depending on the predominant motor phenotype among “probable MSA” group^28^; (2) a clinical follow‐up at least 24 months for the diagnosis confirmation after initial diagnosis; (3) early to moderately advanced stage (disease duration [the period of patient‐reported symptoms until the time of MRI acquisition] <6 years).^18^ Exclusion criteria were: (1) significant cognitive impairment (MMSE < 24); (2) abnormal findings on conventional brain MRI; (3) vascular Parkinson's disease; (4) A history of cerebrovascular disease, neurological surgery; (5) Lack of complete clinical information; (6) Evidence of movement artifacts; (7) Neurological or psychiatric disorders other than PD and MSA (Appendix S1).

The Levodopa equivalent daily dose (LEDD) was calculated.^29^ Motor disability was evaluated using motor scores of the Unified Parkinson's Disease Rating Scale (UPDRSIII). MMSE, Hamilton depression rating scale‐24 items (HDRS‐24), and Hamilton anxiety rating scale (HARS) were assessed. MRI scans were obtained on the same day as the clinical evaluation, with patients during their off‐state (≥12 h after the last reception of dopaminergic medication).

Patients were randomly allocated to the training (70%) and testing (30%) cohort, with stratified sampling. The Institutional Review Board of China Medical University approved this study. Written informed consent was obtained from all patients.

### 2.2 MRI acquisition and preprocessing

The multimodal imaging data were acquired on a 3.0 T MRI scanner (Magnetom Verio, Siemens, Erlangen, Germany) equipped with a 32‐channel head coil. All participants were scanned using a standardized protocol including a high‐resolution T1WI, diffusion tensor imaging (DTI), rs‐fMRI, and SWI sequence. Acquisition parameters are listed in Appendix S2. Quality control was performed by visual inspection. We used FreeSurfer 6.0 software (http://freesurfer.net/; MGH, Boston, MA, USA) to calculate striatal volume. BOLD images were preprocessed using Data Processing and Analysis for Brain imaging (DPABI) software (http://rfmri.org/dpabi). We used advanced normalization tools (ANTs) package for spatial normalization. DTI scalars were obtained using FSL (http://www.fmrib.ox.ac.uk/fsl). The details of preprocessing steps are described in Appendix S3.

### 2.3 Striatum subdivision

Since the striatum is anatomically and functionally segregated, identifying a striatal neuromarker using a fine‐grained striatal parcellation is crucial. Several striatal subdivision methods defined by anatomical or functional alterations are available; however, there is no optimal choice yet. Brainnetome is an anatomically and functionally defined atlas that has been widely applied in neuroimaging studies.^30^, ^31^ Therefore, we selected Brainnetome Atlas for striatal subdivision and brain parcellation. According to the atlas, the striatum contains five subregions per hemisphere: ventral caudate, nucleus accumbens, ventromedial putamen, dorsal caudate, and dorsolateral putamen.

### 2.4 Feature extraction

Regarding rs‐fMRI data, functional activity (regional homogeneity (ReHo) and amplitude of low‐frequency fluctuation [ALFF]) and functional connectivity (intra‐ and extrastriatal FC) were calculated. The details of functional measures extraction are given in Appendix S4. Finally, 10 mALFF, 10 mReHo, 45 intra‐ and 2630 extrastriatal FC values were extracted. Volumes of bilateral striatal subregions were normalized by total intracranial volume. Average values of fractional anisotropy (FA) and mean diffusivity (MD) were calculated in bilateral striatal subregions. In line with the Imaging Biomarker Standardization Initiative (IBSI), 90 radiomic features of each striatal subregion were extracted from SWI^32^, ^33^ (Appendix S5). Ultimately, 3625 striatal‐related functional, structural, iron‐radiomic measures were extracted as features.

### 2.5 Feature selection

Firstly, features were standardized using *z* score normalization. In order to avoid model overfitting, a feature selection procedure was performed to remove redundant features in the training dataset. The mutual information‐based feature selection technique‐minimum redundancy maximum relevance (mRMR) method was applied to select a subset of features with a high correlation with the label and the least correlation among themselves, which, in turn, was applied to scale down the feature vector.^34^ Subsequently, the least absolute shrinkage and selection operator (LASSO) was applied to further eliminate the redundant features. The best hyperparameter lambda of LASSO model was established in accordance with the minimum mean squared error via five‐fold cross‐validation (CV).

### 2.6 Model construction and evaluation

The selected features were applied to the classification model to distinguish IPD from MSA‐P. The support vector machine (SVM) equipped with radial basis function (RBF) is suitable for high‐dimensional data with small sample size and have ability of avoid overfitting by regularization hyperparameters.^35^ Therefore, we applied SVM with RBF kernel for model construction. A nested loop five‐fold CV strategy was applied during the model construction, i.e., the outer loop for model evaluation and the inner loop for optimizing hyperparameter (C and gamma) via the grid‐search method. Based on the SWI, BOLD, DTI, and 3DT1 modalities, we established “Iron,” “Function,” “Diffusion,” and “Volumetry” models, respectively. Additionally, a “multimodal” model based on a combination of four modalities was constructed. Furthermore, we aimed to build a “clinical‐multimodal” model. Clinical variables, which showed statistical difference between groups were considered as valuable and were incorporated into the “clinical‐multimodal” model.

The established models were evaluated on the independent testing cohort. Subgroup validation was further conducted: early stage (disease duration ≤2 years) versus moderately advanced stage (disease duration >2 years). The AUC was applied as the main scalar for model evaluation. Balanced accuracy, sensitivity, specificity, positive predictive value (PPV), and negative predictive value (NPV) were also calculated on both training and testing datasets.

### 2.7 Model interpretation

SHapley Additive exPlanations (SHAP) analysis was conducted to explain the model output and identify the top‐contributing striatal neuromarker for classification.^36^ Regarding the difficulties of explaining the machine learning model, model‐independent SHAP analysis provided insights into the model by calculating the contribution of each feature to the model prediction globally.

### 2.8 Statistical analysis

The demographic and clinical data were analyzed using SPSS software (IBM, Armonk, NY, USA) and MATLAB R2013b. The Kolmogorov–Smirnov test was performed to assess the normal distribution of continuous data. The group comparison between age, disease duration, and UPDRSIII score was carried out using a two‐sample *t*‐test. The mean frame‐wise displacement parameter was compared using Mann–Whitney *U* test between two groups. The differences in gender were compared using the chi‐square test. A permutation test (1000 times) was applied to test the classification accuracy. The DeLong test was used for AUC comparison. Two‐sample *t*‐tests with a false‐discovery rate (FDR) correction were applied to compare selected features between two groups. A two‐sided *p*‐value <0.05 indicated a statistically significant difference.

## 3 RESULTS

### 3.1 Demographic and clinical data

**FIGURE 1:**
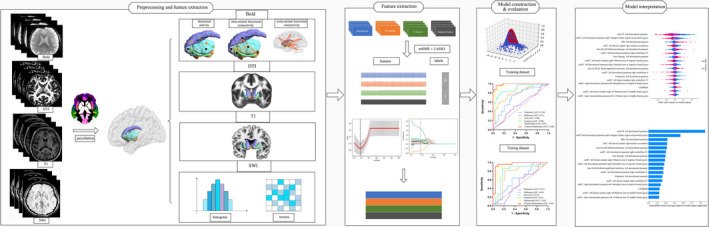
Workflow of the current study. First, the multimodal MRI images (BOLD, DTI, T1 images, and SWI) were obtained and preprocessed. Multimodal striatal scalars were calculated and extracted based on Brainnetome Atlas. Second, features from BOLD, DTI, TI, SWI modalities were selected in the training dataset, which included the minimal redundancy, maximal relevance (mRMR), and least absolute shrinkage and selection operator (LASSO). Third, the support vector machine (SVM) model with a radial basis function (RBF) kernel was constructed to discriminate MSA‐P from IPD. The diagnostic performance was evaluated based on the AUC in the training and testing datasets. Finally, Shapley additive explanations (SHAP) analysis was conducted for multimodal model interpretation.

No significant differences were detected in age, gender, and disease duration (*p* = 0.32, *p* = 0.50, and *p* = 0.16 for training dataset; *p* = 0.37, *p* = 0.30, and *p* = 0.36 for testing dataset) between the two groups. Also, there were no significant differences in MMSE, HDRS‐24, and HARS score (*p* = 0.21, *p* = 0.98, and *p* = 0.62 for training dataset; *p* = 0.36, *p* = 0.73, and *p* = 0.84 for testing dataset) between the two groups. Compared to IPD group, patients in MSA‐P groups showed significant higher UPDRSIII score (*p* = 0.01 for training dataset, *p* = 0.02 for testing dataset). There was no difference in LEDD between IPD and MSA‐P patients (*p* = 0.70 for training dataset, *p* = 0.72 for testing dataset; Table 1). Figure 1 shows the workflow of the current study.

**TABLE 1:** Demographic and clinical characteristics

Characteristics	Training cohort				Testing cohort			
	IPD (*n* = 54)	MSA‐P (*n* = 53)	Analysis		IPD (*n* = 23)	MSA‐P (*n* = 22)	Analysis	
			*T*/*U*/*χ* ^2^	*p*			*T*/*U*/*χ* ^2^	*p*
Age at MRI scan (years)	64.15 ± 6.12	65.32 ± 6.04	−1.00	0.32	63.65 ± 5.53	62.09 ± 5.91	0.92	0.37
Gender (male/female)	25/29	28/25	0.46	0.50	13/10	9/13	1.10	0.30
Disease duration at MRI scan	3.09 ± 1.32	2.74 ± 1.27	1.42	0.16	3.37 ± 1.84	2.89 ± 1.63	0.93	0.36
UPDRSIII score	28.94 ± 12.69	35.60 ± 14.51	−2.53	0.01*	27.39 ± 10.38	34.64 ± 9.29	−2.46	0.02*
MMSE score	27.57 ± 2.10	27.06 ± 2.15	1.26	0.21	27.83 ± 1.97	27.27 ± 2.05	0.92	0.36
HDRS‐24 score	9.50 ± 4.85	9.47 ± 5.31	0.03	0.98	9.48 ± 4.41	9.00 ± 4.78	0.35	0.73
HARS score	10.80 ± 6.30	10.15 ± 7.06	0.50	0.62	10.22 ± 4.71	10.50 ± 4.63	−0.20	0.84
LEDD (mg)	407.26 ± 169.91	421.22 ± 163.42	−0.38	0.70	400.79 ± 157.52	416.47 ± 143.13	0.31	0.72
FD	0.15 (0.11, 0.22)	0.13 (0.09, 0.19)	1233	0.22	0.13 (0.08, 0.22)	0.12 (0.10, 0.19)	167.50	0.85

Abbreviations: FD, mean frame‐wise displacement; HARS, Hamilton anxiety rating scale; IPD, idiopathic Parkinson's disease; LEDD, Levodopa equivalent daily doses; HDRS‐24, Hamilton Depression Rating Scale‐24 items; MMSE, Mini‐Mental state examination; MSA‐P, parkinsonian variant of multiple system atrophy; *SD*, standard deviation; UPDRSIII, the unified Parkinson's Disease Rating Scale.

* Statistical significance *p* < 0.05.

### 3.2 Feature selection

**FIGURE 2:**
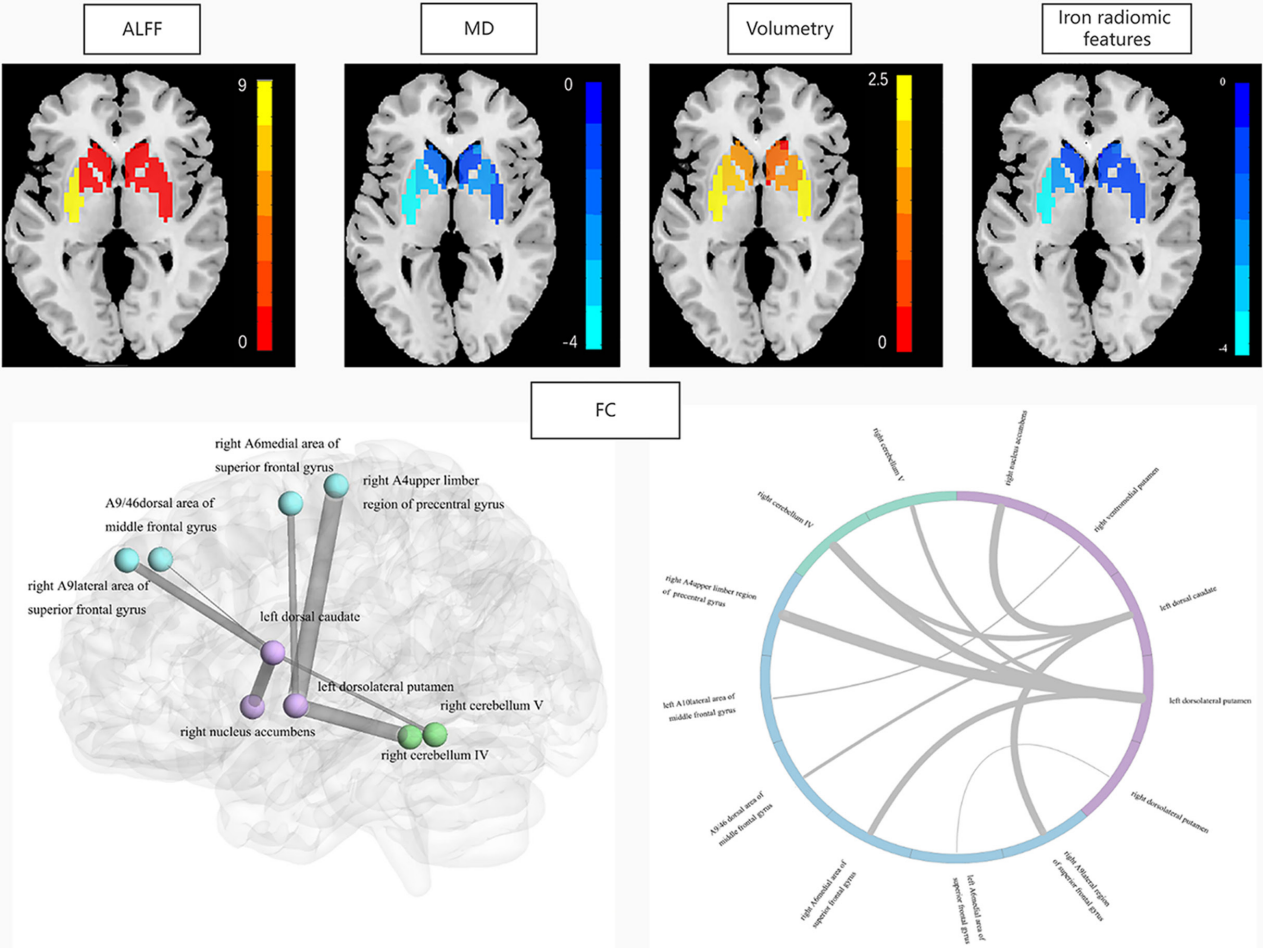
Selected features in the Multimodal SVM model. The amplitude of low‐frequency fluctuation (ALFF), mean diffusivity (MD) value, the volume, and the iron‐radiomic features of the left dorsolateral putamen were selected (upper). The extrastriatal and intrastriatal functional connectivity (FC) in the “Multimodal” model (below). The thickness of the links in the FC data represents the weight of significant connections according to SHAP analysis results in “Multimodal” model.

As for “multimodal” model, 3625 features were initially reduced to 55 potential variables after mRMR procedure, which included 31 extrastriatal FC, 3 intrastriatal FC, 4 mALFF, 2 mReHo, 2 FA, 2 MD, 3 volume, and 8 iron‐radiomic features. Subsequently, 16 features with nonzero coefficients in the LASSO model were selected in the training dataset: 9 extrastriatal FC, 1 intrastriatal FC, 1 mALFF, 1 MD, 1 volume, and 3 iron‐radiomic features (Figure 2).

Specifically, the mALFF value of the left dorsolateral putamen (0.71 ± 0.12 in IPD; 0.43 ± 0.18 in MSA‐P, *p* < 0.001) in MSA‐P patients was lower than that in IPD patients. The extrastriatal FC of the dorsal striatum and frontal lobe, as well as the cerebellum were found to be comparatively lower in MSA‐P patients, compared with that in IPD patients. Similarly, the volume of the left dorsolateral putamen (0.20 ± 0.02 in IPD; 0.20 ± 0.03 in MSA‐P, *P* = 0.018) was lower in MSA‐P patients than that in IPD patients. Conversely, the MD value of the left dorsolateral putamen (0.08 ± 0.01 in IPD; 0.09 ± 0.02 in MSA‐P, *p* < 0.001) and iron‐related radiomic features (IPD: 1.23 ± 0.20 for DifferenceEntropy, 1.96 ± 0.38 for Entropy, 0.38 ± 0.07 for RunLengthNonUniformityNormalized; MSA‐P: 1.43 ± 0.29 for DifferenceEntropy, 2.30 ± 0.48 for Entropy, 0.43 ± 0.07 for RunLengthNonUniformityNormalized, *p* < 0.01) were higher in MSA‐P patients than those in IPD patients (Table 2). The details of the selected features in each model are presented in Appendix S6. Besides, only UPDRSIII score showed statistical difference between IPD and MSA‐P patients (*p* < 0.05) among clinical variables. Therefore, UPDRSIII was further included in the “clinical‐multimodal” model.

**TABLE 2:** Characteristics of the selected features in the “multimodal,” “Function,” “Diffusion,” “Volumetry,” “Iron” models

Model	Features	IPD	MSA‐P	Adjusted‐*p*
Multimodal	mALFF: left dorsolateral putamen	0.71 ± 0.12	0.43 ± 0.18	<0.001***
	outFC: left dorsolateral putamen‐right A4 upper limber region of precentral gyrus	0.38 ± 0.17	0.21 ± 0.13	<0.001***
	MD (×100): left dorsolateral putamen	0.08 ± 0.01	0.09 ± 0.02	<0.001***
	outFC: left dorsolateral putamen‐right cerebellum IV	0.26 ± 0.15	0.16 ± 0.12	<0.001***
	inFC: left dorsal caudate‐right nucleus accumbens	0.41 ± 0.17	0.29 ± 0.14	<0.001***
	Iron GLCM‐DifferenceEntropy: left dorsolateral putamen	1.23 ± 0.20	1.43 ± 0.29	<0.001***
	Iron Entropy: left dorsolateral putamen	1.96 ± 0.38	2.30 ± 0.48	<0.001***
	outFC: left dorsal caudate‐right A9 lateral region of superior frontal gyrus	0.47 ± 0.19	0.36 ± 0.20	<0.001***
	outFC: left dorsolateral putamen‐right A6 medial area of superior frontal gyrus	0.28 ± 0.16	0.18 ± 0.15	0.002**
	outFC: left dorsal caudate ‐right cerebellum IV	0.34 ± 0.17	0.24 ± 0.21	0.009**
	Iron GLRLM‐GLRLM‐RunLengthNonUniformityNormalized: left dorsolateral putamen	0.38 ± 0.07	0.43 ± 0.07	0.002**
	OutFC: left dorsolateral putamen‐right cerebellum V	0.32 ± 0.17	0.21 ± 0.17	0.012*
	OutFC: left dorsal caudate‐right A9/46 dorsal area of middle frontal gyrus	0.34 ± 0.19	0.24 ± 0.16	0.005**
	OutFC: right ventromedial putamen‐left A10 lateral area of middle frontal gyrus	0.32 ± 0.12	0.26 ± 0.13	0.003**
	OutFC: right dorsolateral putamen‐left A6medial area of superior frontal gyrus	0.27 ± 0.17	0.19 ± 0.12	0.011*
	Volumetry (×100): left dorsolateral putamen	0.20 ± 0.02	0.20 ± 0.03	0.018*
Function	mALFF: left dorsolateral putamen	0.71 ± 0.12	0.43 ± 0.18	<0.001***
	OutFC: left dorsolateral putamen‐right A4 upper limber region of precentral gyrus	0.38 ± 0.17	0.21 ± 0.13	<0.001***
	OutFC: left dorsolateral putamen‐right cerebellum IV	0.26 ± 0.15	0.16 ± 0.12	0.001***
	InFC: left dorsal caudate‐right nucleus accumbens	0.41 ± 0.17	0.29 ± 0.14	<0.001***
	outFC: left dorsal caudate‐right A9 lateral region of superior frontal gyrus	0.47 ± 0.19	0.37 ± 0.20	0.010*
	outFC: left dorsolateral putamen‐right A6medial area of superior frontal gyrus	0.28 ± 0.16	0.18 ± 0.148	0.002**
	outFC: left dorsal caudate ‐right cerebellum IV	0.34 ± 0.17	0.24 ± 0.21	0.010*
	outFC: left dorsolateral putamen‐right cerebellum V	0.32 ± 0.17	0.21 ± 0.17	0.005**
	outFC: left dorsal caudate‐right A9/46dorsal area of middle frontal gyrus	0.34 ± 0.19	0.24 ± 0.16	0.007**
	outFC: right ventromedial putamen‐left A10lateral area of middle frontal gyrus	0.32 ± 0.12	0.26 ± 0.13	0.010*
	outFC: right dorsolateral putamen‐left A6medial area of superior frontal gyrus	0.27 ± 0.17	0.19 ± 0.12	0.010*
	outFC: left dorsolateral putamen‐left lateral prefrontal thalamus	0.36 ± 0.18	0.27 ± 0.18	0.010*
Diffusion	MD (×100): left dorsolateral putamen	0.08 ± 0.01	0.09 ± 0.02	<0.001***
	FA: left dorsal caudate	0.25 ± 0.03	0.23 ± 0.03	0.012*
Volumetry	Volume (×100): left dorsolateral putamen	0.20 ± 0.02	0.20 ± 0.03	0.007**
	Volume (×100): right dorsolateral putamen	0.20 ± 0.02	0.20 ± 0.03	0.013*
Iron	Entropy: left dorsolateral putamen	1.96 ± 0.38	2.30 ± 0.48	<0.001***
	DifferenceEntropy: left dorsolateral putamen	1.23 ± 0.20	1.43 ± 0.29	<0.001***
	GLRLRunLengthNonUniformityNormalized: left dorsolateral putamen	0.38 ± 0.07	0.43 ± 0.07	0.001**
	GLSZM‐SizeZoneNonUniformityNormalized: left dorsolateral putamen	0.24 ± 0.04	0.27 ± 0.05	0.009**

*Note*: Values expressed as mean ± *SD*.

Abbreviations: FA, fractional aniotrophy; GLCM, gray level co‐occurrence matrix; GLRLM, gray level run length matrix; GLSZM, gray level size zone matrix; IPD, idiopathic Parkinson's disease; MD, mean diffusivity; MSA‐P, parkinsonian variant of multiple system atrophy.

*Statistical significance, *p* < 0.05; **statistical significance, *p* < 0.01; ***statistical significance, *p* < 0.001.

### 3.3 Model performance

**FIGURE 3:**
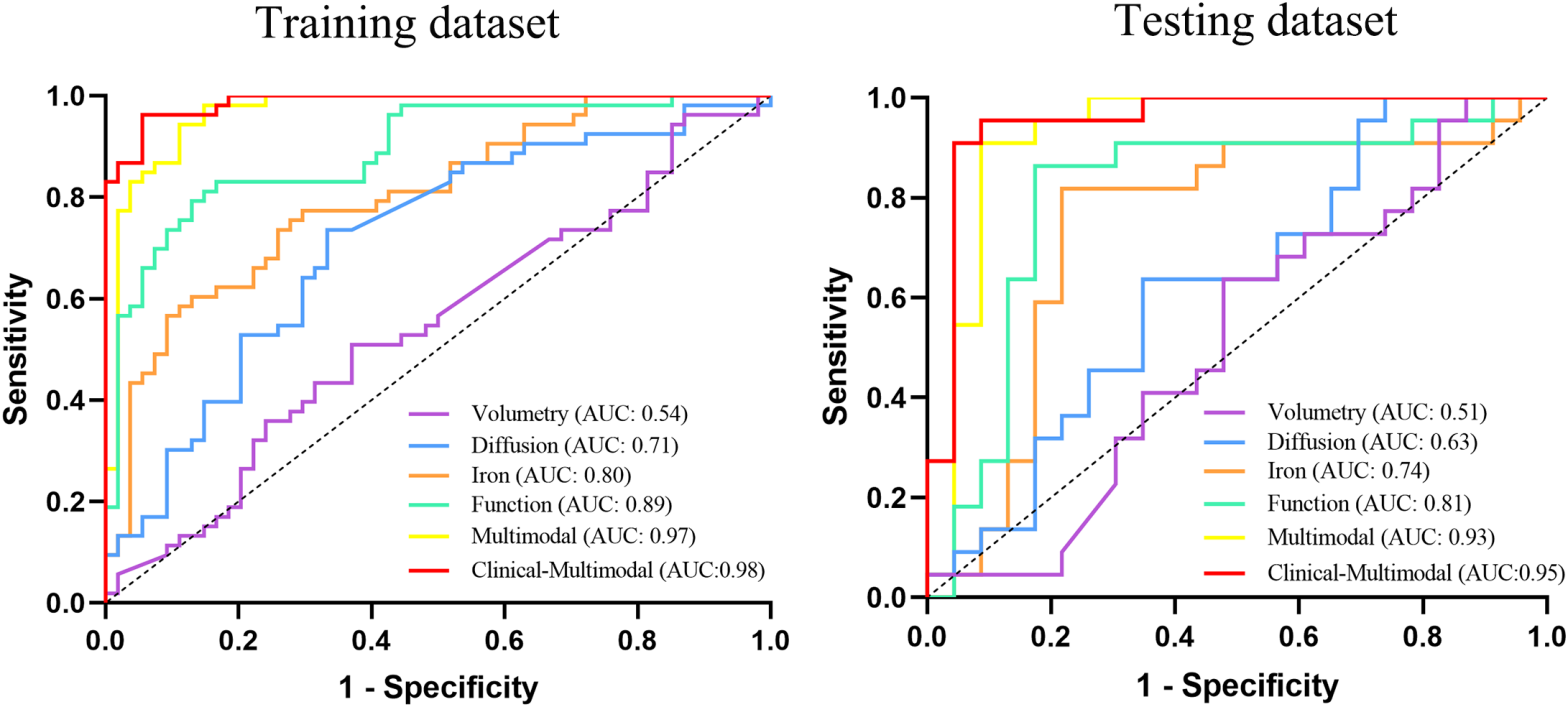
Receiver operator characteristic curve (ROC) of the “Function,” “Diffusion,” “Volumetry,” "Iron," “Multimodal,” and “Clinical‐Multimodal” models in the training (left) and testing dataset (right).

In the training and testing cohort, the “Multimodal” model yielded the best classification performance, with AUC of 0.968 (0.914–0.992) and 0.927 (0.809–0.983), respectively. The performance of “Function,” “Iron,” “Diffusion,” and “Volumetry” models were moderate to poor, in decreasing order, with AUC of 0.890 (0.815–0.942), 0.800 (0.712–0.871), 0.713 (0.618–0.797), and 0.544 (0.445–0.641) in the training dataset and 0.806 (0.661–0.909), 0.741 (0.589–0.860), 0.626 (0.470–0.766), and 0.514 (0.360–0.665) in the testing dataset, respectively. The “Clinical‐Multimodal” model further increased the performance, yielding an AUC of 0.986 (0.944–0.999) and 0.953 (0.885–0.994; Table 3 and Figure 3). Furthermore, the “multimodal” and “clinical‐multimodal” model had slightly better performance in the moderately advanced subgroup relative to the early‐stage subgroup, despite not surviving the Delong test (Appendix S7).

**TABLE 3:** The performance of “Function,” ”Diffusion,” ”Volumetry,” “Iron,” ”Multimodal,” and “clinical‐multimodal” model in the differential diagnosis

Model	Training dataset						Testing dataset					
	AUC (95% CI)	B‐ACC	Sen	Spec	PPV	NPV	AUC (95% CI)	B‐ACC	Sen	Spec	PPV	NPV
Function	0.890 (0.815–0.942)	0.832*	0.833	0.830	0.830	0.833	0.806 (0.661–0.909)	0.845*	0.864	0.826	0.826	0.864
Diffusion	0.713 (0.618–0.797)	0.702*	0.736	0.667	0.684	0.720	0.626 (0.470–0.766)	0.644*	0.636	0.652	0.636	0.652
Volumetry	0.544 (0.445–0.641)	0. 570*	0.509	0.630	0.574	0.567	0.514 (0.360–0.665)	0.579*	0.636	0.522	0.560	0.600
Iron	0.800 (0.712–0.871)	0.740*	0.774	0.704	0.719	0.760	0.741 (0.589–0.860)	0.801*	0.818	0.783	0.783	0.818
Multimodal	0.968 (0.914–0.992)	0.917*	0.981	0.852	0.867	0.979	0.927 (0.809–0.983)	0.911*	0.909	0.913	0.909	0.913
Clinical‐Multimodal	0.986 (0.944–0.999)	0.963*	0.981	0.944	0.945	0.981	0.953 (0.885–0.994)	0.934*	0.955	0.913	0.913	0.955

Abbreviations: AUC, area under the receiver operator curve; B‐ACC, balanced accuracy; Sen, sensitivity; Spec, specificity; PPV, positive predict value; NPV, negative predict value.

* *p* < 0.001 under permutation test (1000 times).

Delong tests results showed that the “Clinical‐Multimodal” model outperformed “Function” model (*p* = 0.003), “Iron” model (*p* < 0.001), “Diffusion” model (*p* < 0.001), and “Volumetry” model (*p* < 0.001) in the training dataset, as well as “Iron” model (*p* = 0.016), “Diffusion” model (*p* < 0.001), and “Volumetry” model (*p* < 0.001) in the testing dataset. Similarly, the “Multimodal” model outperformed “Function” model (*p* = 0.036), “Iron” model (*p* < 0.001), “Diffusion” model (*p* < 0.001), and “Volumetry” model (*p* < 0.001) in the training dataset, as well as “Diffusion” model (*p* = 0.003), and “Volumetry” (*p* < 0.001) model in the testing dataset. As for models of single modality, the “Function” model outperformed the “Diffusion” model (*p* = 0.002) and “Volumetry” (*p* < 0.001) model in the training dataset, as well as the “Volumetry” model (*p* = 0.015) in the testing dataset (Appendix S8). The optimized hyperparameters of the models are shown in Appendix S9.

### 3.4 Model interpretation with SHAP

**FIGURE 4:**
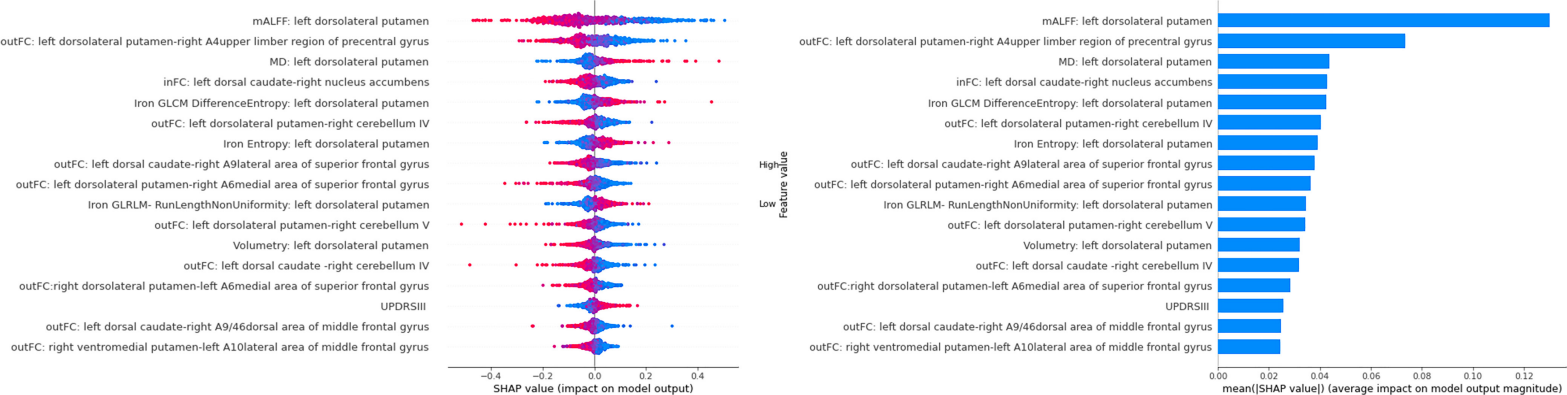
Shapley additive explanations summary plot of features of the “Clinical‐Multimodal” model. Left: SHAP values of features for every sample. Each line represents a feature, each dot represents a sample. Red represents higher feature values, blue represents lower feature values. Right: mean absolute SHAP values of features sorted in descending rank order. Abbreviations: inFC, intrastriatal functional connectivity; mALFF, mean amplitude of low‐frequency fluctuation; MD, mean diffusivity; outFC, extrastriatal functional connectivity; UPDRSIII, Motor scores of the unified Parkinson's Disease Rating Scale.

In the “Multimodal” and “Clinical‐Multimodal” model prediction, the mALFF value of the left dorsolateral putamen was a critical predictor, with a decrease of mALFF in the left dorsolateral putamen corresponding to increased confidence in the MSA‐P diagnosis. The FC between the left dorsolateral putamen and the right precentral gyrus is a major factor in the model prediction. (Figure 4 and Appendix S10). The mALFF, MD value, volume, and Difference Entropy of the left dorsolateral putamen is a vital predictor in “Function,” “Diffusion,” “Volumetry,” and “Iron” models, respectively (Appendices [Link], [Link], [Link], [Link]).

## 4 DISCUSSION

In this study, the machine learning algorithm identified the striatal dysfunction pattern with an excellent performance characterized by multimodal MRI distinguishing patients with IPD and MSA‐P in clinical settings. Notably, the model exhibited good performance and was well‐interpretable, manifesting the dorsolateral putamen as the most valuable neuromarker for classification. The dorsal striatum dysfunction might further cause supervening frontal lobe and cerebellar dysfunction through frontostriatal and cerebello‐striatal circuits, leading to clinical motor and cognitive symptoms.

Identifying the diagnostic biomarker for classification between IPD and MSA is currently underway. For example, clonidine and arginine growth hormone stimulation test can differentiate MSA from IPD. The measures of the growth hormone response to clonidine and arginine in serum sample serves as in indirect indicator for the function of various neurotransmitter networks. Previous studies reported a high differential diagnosis performance, ranging from 73.08% to 96%.^37^, ^38^, ^39^ Despite its high diagnostic performance, the test may be restricted to clinical practice due to its confounded effect by dopaminergic treatment and its invasive procedure. Therefore, non‐invasive differential diagnostic biomarker is warranted. Neuroimaging method has been introduced as an effective non‐invasive approach for classification. However, studies focusing on the differential diagnosis between IPD and MSA‐P are sparse. Previous studies revealed the putamen's role in the differential diagnosis.^40^, ^41^ Since the findings were population‐based, the ability to provide valuable evidence at the individual level is less well‐established. Recent studies applied discriminant analysis and machine learning algorithms for differential diagnosis.^21^, ^23^, ^24^, ^26^ Nonetheless, with small sample size and no separate independent testing dataset, the models might be overfitting with a lack of generalization. In addition, functional MRI modality was not included but might capture brain damage at an early stage. In order to transpose an unbiased and high accuracy classification model, we improved the above limitations. Firstly, the number of MSA‐P patients is large in the current study but limited compared to previous studies. Secondly, the information of striatal dysfunction depicted by iron‐sensitive, functional, and structural multimodal MRI renders it to a high accuracy model. Thirdly, with an independent testing dataset, the model has high accuracy in the training dataset and is generalized satisfactorily to the testing dataset. With high generalization and robustness, the model may have clinical potential. Finally, since the process of striatal segmentation and multimodal neuroimaging feature extraction was automatic, our study provides an automatic pipeline for the classification of IPD and MSA‐P individuals.

Furthermore, the multiscale characterization of striatum dysfunction provided by multimodal iron‐sensitive, functional, and structural MRI was superior to either of these modalities used independently for the differential diagnosis of IPD and MSA‐P. Some studies also indicated that biomarkers from various modalities provide complementary information, thereby improving the performance compared to the methods based on single modal data.^42^ Besides, “clinical‐multimodal” model, which incorporate motor severity into the multimodal model, could further increase the diagnostic performance. The result highlights the importance of a combination of neuroimaging examinations and clinical measurement in making clinical diagnosis. Patients with MSA had more severe motor impairment than did those with IPD, underlying the role of UPDRSIII as a potential differential indicator.^1^ Furthermore, the “multimodal” and “clinical‐multimodal” models showed slightly better performance in moderately advanced subgroup compared with very early subgroup, despite not statistically conclusive. The possible explanation may be that disease specific abnormalities could not be captured by multimodal MRI until the moderately advanced stage of the disease. As for the single modality models, the “Function model” performance was superior to “Iron model,” followed by “Diffusion model” and “Volumetry model.” Functional MRI can unravel disease mechanisms from an early stage based on the functional correlation of regenerative neuronal cell death,^43^ while brain macrostructural changes reflect irreversible neuronal cell loss and can be observed in moderate to severe patients, providing incomplete information of the disease.

Based on SHAP analysis results, the dorsolateral putamen served as a critical neuromarker in the classification of IPD and MSA‐P. Accumulating evidence on iron‐sensitive images with respect to a sign of putaminal hypointensity from lateral to medial in MSA‐P patients suggested severe iron accumulation in the lateral putamen.^6^, ^44^ Radiomic features could reflect the pattern of local iron deposition by depicting local homogeneity. In our study, iron accumulation of the dorsolateral putamen was more complex in MSA‐P patients compared with IPD patients. Elevated focal iron further triggers cellular and tissue damage by stimulating a‐synuclein aggregation,^45^ which can be detected by rs‐fMRI at an early stage. Thus, compared to IPD, individuals with MSA‐P manifested decreased functional activity in the dorsolateral putamen. The elevated putaminal MD values in MSA‐P could be attributed to the focal tissue architecture destroyed by neuronal degeneration.^46^

Taken together, the functional connectivity between the dorsal striatum and frontal lobule, as well as dorsal striatum and cerebellum, plays a key role in differential diagnosis. The symptoms of parkinsonian diseases are not associated with dysfunction of isolated striatum, but are related to a cascade of pathophysiological alterations in multiple brain regions via neuronal circuits.^47^ The dorsal striatum consists of the putamen and caudate and controls the motor and cognitive function by cortico‐striatal and cerebello‐striatal circuits.^48^, ^49^, ^50^ Specifically, the dorsolateral putamen is associated with motor function giving rise to the motor and premotor cortices via the frontostriatal loop.^51^ Besides, our previous work revealed the relationship between anterior cerebellum and motor symptoms in MSA patients.^52^ In the current study, the reduced functional connectivity between the dorsolateral putamen and prefrontal gyrus, as well as dorsolateral putamen and anterior cerebellum were found in the MSA‐P group. The decreased functional connectivity in MSA‐P might provide explanation for the nature of more severe clinical symptoms reported in MSA group according to previous studies.^1^ Conversely, the dorsal caudate is involved in executive function and working memory via connectivity to the dorsolateral prefrontal cortex. In line with this phenomenon, the functional connectivity of dorsal caudate and superior and middle frontal gyrus was reduced in the MSA‐P group compared to IPD patients. The decreased functional connectivity between dorsal caudate and prefrontal gyrus might cause a more severe cognitive impairment in MSA‐P than PD patients.^53^, ^54^ In addition, the preserved functional connectivity of dorsal striatum and frontal lobe in IPD may be relevant to the functional modulatory mechanism, representing a network response to the local neuronal injury in order to maintain the global performance.^55^ Overall, the dysfunction in the dorsal striatum, especially dorsolateral putamen, caused supervening prefrontal lobe and cerebellum dysfunction via the frontostriatal and cerebello‐striatal circuit, which resulted in motor and cognitive symptoms.

Intriguingly, the left side of striatal dysfunction was more dominant than the right side. Dopamine transporter single‐photon emission computed tomography (DAT‐SPECT) detected a trend of left hemispheric predominance of nigrostriatal deficits in right‐handed PD patients.^56^ Also, motor symptoms were observed in the right side of the body in right‐handed PD.^57^ Therefore, we speculated that the left side of the striatum was deprived of dopamine supplement, followed by local functional activity dysfunction, which then translated to the contralateral cortical dysfunction through the striatal‐cortical loop, finally resulting in motor symptoms in the contralateral side. However, the asymmetry of nigrostriatal deficits needs further investigation.

Nevertheless, this study has several limitations. First, there is a lack of neuropathological confirmation of IPD and MSA diagnosis, which is also observed for the majority of neurodegenerative studies. Second, neural differences under the similar disease duration could be greater in MSA‐P patients compared with IPD, which might have influence on the model performance. Third, the sample size in the current study is not that large, but still larger than previous studies, necessitating a large multicenter cohort study. In addition, the optimal strategy for striatal subdivisions is yet lacking. Thus, a comparison of models using different parcellation approaches is required. Finally, in view of clinical‐multimodal model construction, we used UPDRSIII to evaluate motor severity in MSA patients, which may be not a specific rating scale for MSA. Besides, further studies on determining whether established models correlate with the various parts of UPDRS is warranted.

## 5 CONCLUSIONS

In summary, multimodal striatal dysfunction, which mainly involved the dorsal striatum and its supervening prefrontal lobe and cerebellum dysfunction via the frontostriatal and cerebello‐striatal circuit, contributed to the classification of IPD and MSA‐P and further facilitated individualized treatment in clinical settings. A combination of multimodal striatal neuromarkers and UPDRIII score could further improve diagnostic performance. Among the dorsal striatum subregions, the dorsolateral putamen can be deemed the most valuable neuromarker for differential diagnosis.

## AUTHOR CONTRIBUTIONS

Dr Guoguang Fan involved in the conceptualization and supervision of the study. Huize Pang involved in analysis and interpretation of data, drafting the article. Ziyang Yu involved in data methodology. Hongmei Yu involved in data validation. Miao Chang reviewed the manuscript. JiBin Cao, Yingmei Li, Miaoran Guo, Yu Liu, Kaiqiang Cao involved in the acquisition of data. We thank MedSci Healthcare for its linguistic assistance during preparation of this manuscript.

## FUNDING INFORMATION

This study was supported by research grants from the National Science Foundation of China (82071909).

## CONFLICTS OF INTEREST

The authors declare that they have no competing interests.

## Supporting information


Appendix S1

Appendix S2

Appendix S3

Appendix S4

Appendix S5

Appendix S6

Appendix S7

Appendix S8

Appendix S9

Appendix S10

Appendix S11

Appendix S12

Appendix S13

Appendix S14

## Data Availability

The data that support the findings of this study are available from the corresponding author upon reasonable request.
