# Supplementary figures and images for: Multimodal striatal neuromarkers in distinguishing parkinsonian variant of multiple system atrophy from idiopathic Parkinson's disease
Source: CNS Neurosci Ther. 2022 Sep 1;28(12):2172–82. doi: 10.1111/cns.13959 (PMC9627351; doi:10.1111/cns.13959)

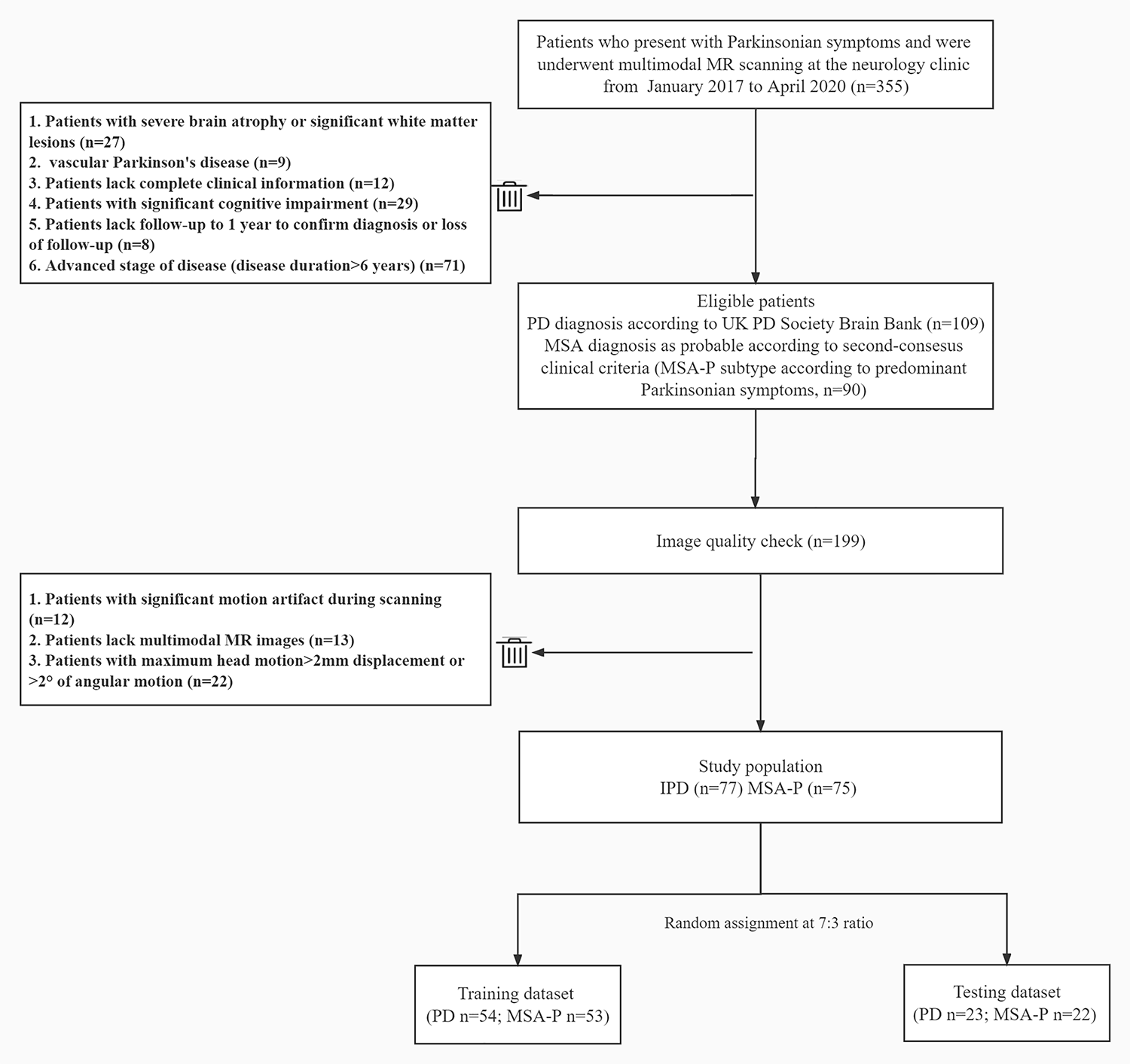

Supplement: Supplementary file 1 — Appendix S1 [file CNS-28-2172-s014.tif]

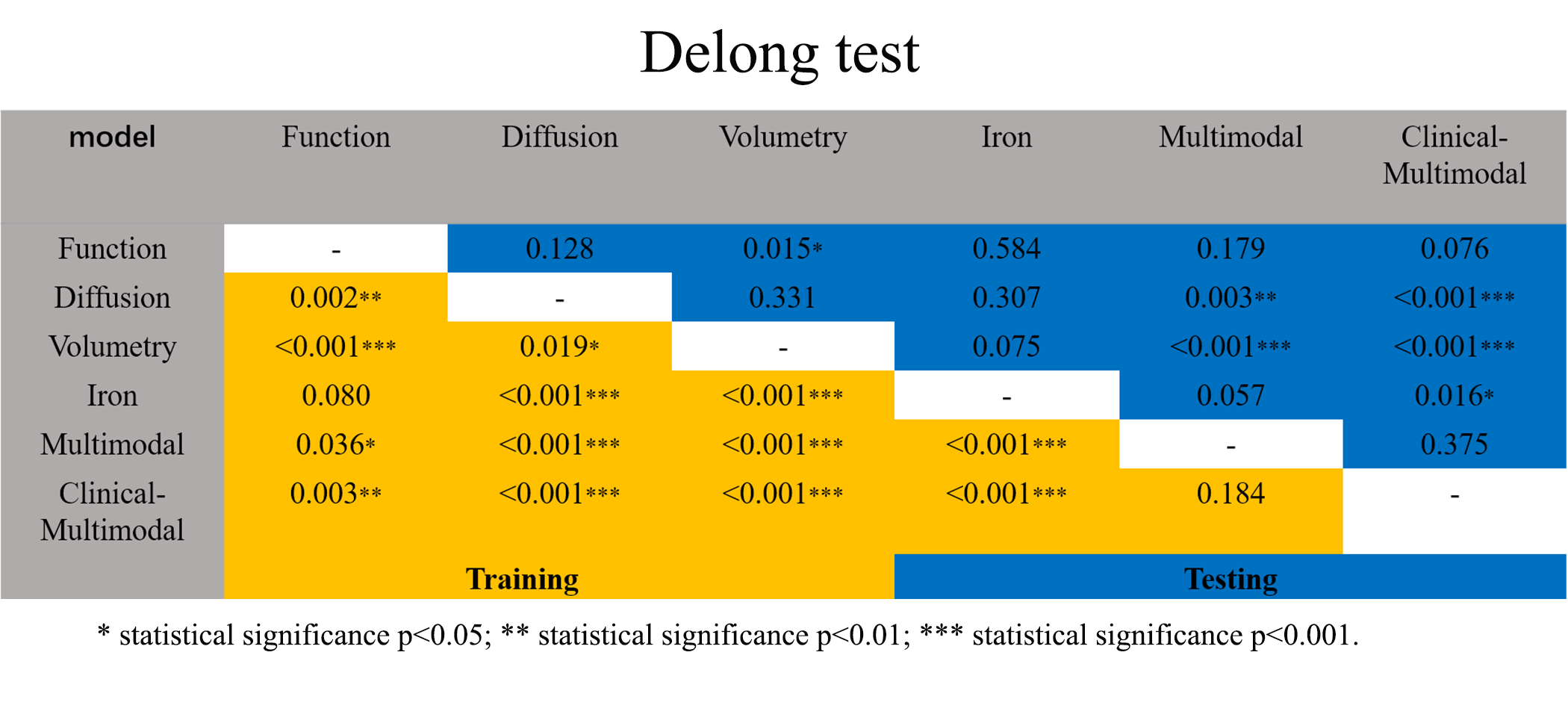

Supplement: Supplementary file 8 — Appendix S8 [file CNS-28-2172-s002.tif]

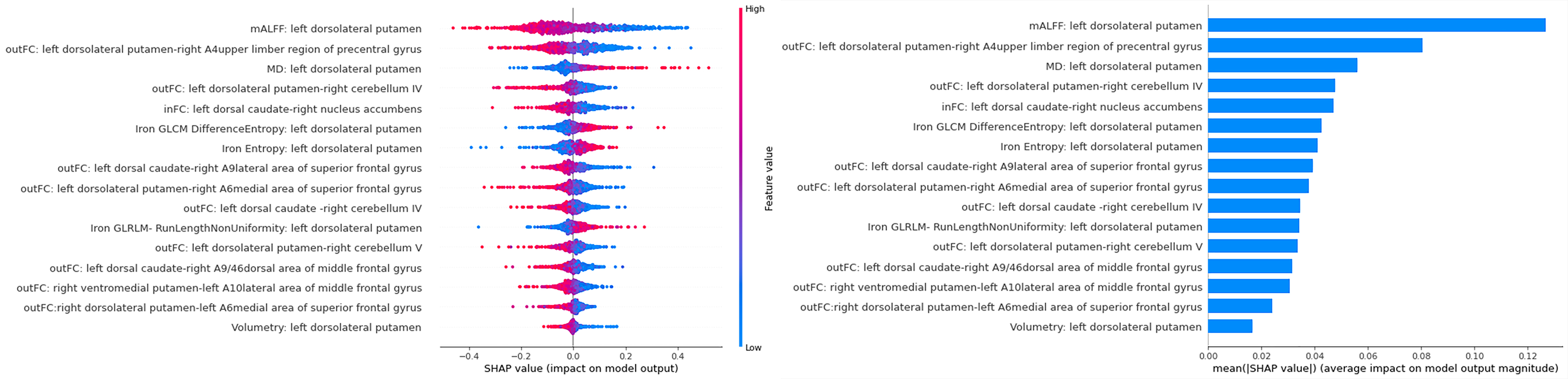

Supplement: Supplementary file 10 — Appendix S10 [file CNS-28-2172-s005.tif]

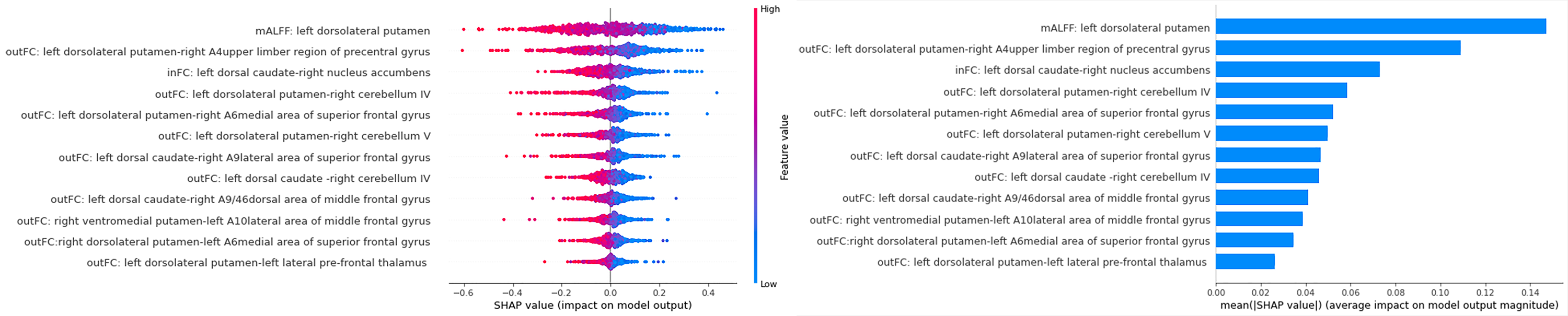

Supplement: Supplementary file 11 — Appendix S11 [file CNS-28-2172-s001.tif]

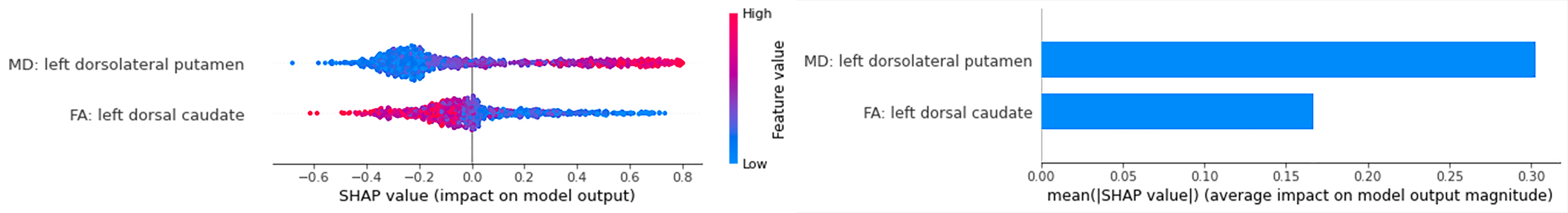

Supplement: Supplementary file 12 — Appendix S12 [file CNS-28-2172-s010.tif]

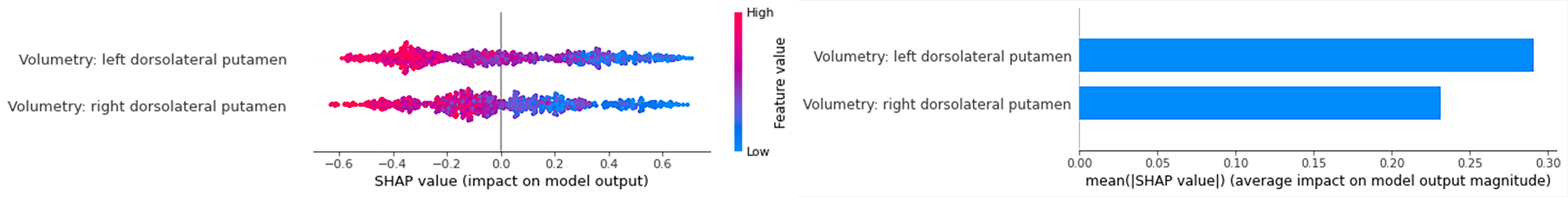

Supplement: Supplementary file 13 — Appendix S13 [file CNS-28-2172-s006.tif]

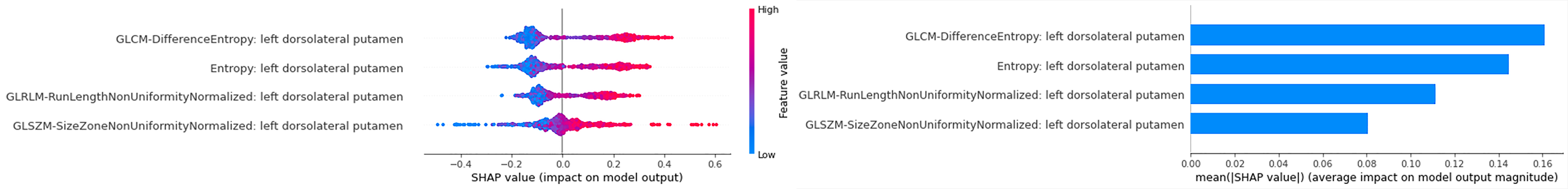

Supplement: Supplementary file 14 — Appendix S14 [file CNS-28-2172-s011.tif]
