# Supplementary material for: Multimodal striatal neuromarkers in distinguishing parkinsonian variant of multiple system atrophy from idiopathic Parkinson's disease
Source: CNS Neurosci Ther. 2022 Sep 1;28(12):2172–82. doi: 10.1111/cns.13959 (PMC9627351; doi:10.1111/cns.13959)
Supplement: Supplementary file 2 — Appendix S2 [file CNS-28-2172-s012.docx]

Image parameters:

1. High resolution three dimensional structural T1-weighted (3DT1) sequence: TE = 2960 ms, TR = 5000ms, flip angle = 12^o^, field of view = 256×256 mm^2^, matrix size = 256×256, slice thickness = 1mm, voxel size = 1.0×1.0×1.0mm.
2. Resting-state functional (rs-fMRI) sequence: TE = 30 ms, TR = 2500 ms, flip angle = 90^o^, field of view = 224×224 mm^2^, matrix size = 64×64, slice thickness = 3.5 mm, voxel size = 3.5×3.5×3.5 mm.
3. Diffusion tensor imaging (DTI) sequence: TE = 95 ms, TR = 10300 ms, field of view = 256×256 mm^2^, matrix size = 128×128, slice thickness = 2 mm, voxel size = 2.0×2.0×2.0mm, number of directions = 64, b value = 1000 sec/mm^2^, b0 value = 0 sec/mm^2^.
4. Susceptibility weighted imaging (SWI) sequence: TE = 20 ms, TR = 27ms, flip angle = 15^o^, field of view = 230×172.5 mm^2^, matrix size = 182×256, slice thickness = 0.8mm, voxel size = 0.9×0.9×0.8mm.
