# Supplementary material for: Multimodal striatal neuromarkers in distinguishing parkinsonian variant of multiple system atrophy from idiopathic Parkinson's disease
Source: CNS Neurosci Ther. 2022 Sep 1;28(12):2172–82. doi: 10.1111/cns.13959 (PMC9627351; doi:10.1111/cns.13959)
Supplement: Supplementary file 3 — Appendix S3 [file CNS-28-2172-s008.docx]

Image preprocessing:

1. Rs-fMRI data were preprocessed using Data Processing Assistant for Resting-state fMRI (DPABI) software (<http://rfmri.org/dpabi>) in Matlab (R2013b; The MathWorks, Inc., Natick, MA, USA) as follows: a) removal of first ten time points; b) slice timing for interval scanning; c) head motion correction (Subjects with head motion >2 mm maximum displacement or >2° of angular motion were excluded) ; d) spatial normalization to the standard Montreal Neurological Institute (MNI) space using advanced normalization tools (ANTs); e) detrending; f) linear regression of Friston 24-head motion parameters, white matter, and cerebrospinal fluid signals; g) temporal bandpass filtering (0.01–0.08 Hz); h) smoothing with a 6-mm full-width at half maximum.

( note : ReHo: preprocessing steps: a–g; ALFF preprocessing steps: a–f, h; functional connectivity: preprocessing steps: a–h).

1. 3DT1 images were automatically segmented using FreeSurfer 6.0 (<http://freesurfer.net/>; MGH, Boston, MA, USA). The main steps were as follows: a) skull stripping; b) Talairach space registration; c) bias field correction; d) GM, white matter (WM), and cerebrospinal fluid segmentation; e) tessellation of GM and WM boundary; f) quality control and manual correction; g) surface deformation and inflation; h) spherical atlas registration; i) mapping Brainnetome Atlas in the freesurfer space of each subject based on prior probability files; j) striatal subregions volume and total intracranial volume calculation.
2. DTI data were processed using FSL tools (<http://www.fmrib.ox.ac.uk/fsl>), including a) Motion and eddy currents correction; b) non-weighted image (b0) extraction; c) registration to the corresponding b0 images; d) brain tissue extraction; e) fitting diffusion tensor model; f) co-registration of Brainnetome Atlas to the individual diffusivity maps using linear and non-linear registration methods and nearest-neighbor interpolation (FLIRT and FNIRT in FSL).
3. Magnitude images of SWI sequence were firstly nonlinearly transformed into standard MNI space using ANTs package. The realigned magnitude images were then separated by Brainnetome templates and ten volumes of interest (VOI) of striatal subregions were obtained. Consistent with the Imaging Biomarker Standardization Initiative (IBSI), 90 radiomic features of each striatal subregions were extracted from SWI sequence, comprising 18 first-order features, 21 grey-level co-occurrence matrix (GLCM) features, 14 grey-level dependence matrix (GLDM) features, 16 grey-level run length matrix (GLRLM) features, 16 grey-level size zone matrix (GLSZM) features, and 5 neighboring grey tone difference matrix (NGTDM) features by using Pyradiomics (<http://www.radiomics.io/pyradiomics.html>).
