# Supplementary material for: Multimodal striatal neuromarkers in distinguishing parkinsonian variant of multiple system atrophy from idiopathic Parkinson's disease
Source: CNS Neurosci Ther. 2022 Sep 1;28(12):2172–82. doi: 10.1111/cns.13959 (PMC9627351; doi:10.1111/cns.13959)
Supplement: Supplementary file 4 — Appendix S4 [file CNS-28-2172-s013.docx]

The mean ReHo and mean ALFF maps were generated following rs-fMRI preprocessing, and the values were extracted by averaging all the voxels within bilateral segmented striatal subregions. Intra- and extrastriatal FC values were obtained by calculating Pearson’s correlation between the time series of each pair of striatal regions of interest (ROIs) and between the time series of each striatal ROI and all other cortical and subcortical ROIs, respectively, followed by z-transformation of correlation coefficients. Considering the cerebellar vermis crus I is too minute to observe under the 3mm^3^ voxel size on rs-fMRI, the FC between striatal subregions and cerebellar vermis crus I was excluded for the analysis.
