# Supplementary material for: Multimodal striatal neuromarkers in distinguishing parkinsonian variant of multiple system atrophy from idiopathic Parkinson's disease
Source: CNS Neurosci Ther. 2022 Sep 1;28(12):2172–82. doi: 10.1111/cns.13959 (PMC9627351; doi:10.1111/cns.13959)
Supplement: Supplementary file 5 — Appendix S5 [file CNS-28-2172-s009.docx]

| Radiomic features of each striatal subregion extracted from SWI sequence | | |
| --- | --- | --- |
| Type | Features | Number |
| First-order | 10Percentile, 90Percentile, Energy, Entropy, InterquartileRange, Kurtosis, Maximum, MeanAbsoluteDeviation, Mean, Median, Minimum, Range, RobustMeanAbsoluteDeviation, RootMeanSquared, Skewness, TotalEnergy, Uniformity, Variance | 18 |
| GLCM | Autocorrelation, ClusterProminence, ClusterShade, ClusterTendency, Contrast, Correlation, DifferenceAverage, DifferenceEntropy, DifferenceVariance, Id, Idm, Idmn, Idn, Imc1, Imc2, InverseVariance, JointAverage, JointEnergy, JointEntropy, MaximumProbability, SumEntropy | 21 |
| GLDM | DependenceEntropy, DependenceNonUniformity, DependenceNonUniformityNormalized, DependenceVariance, GrayLevelNonUniformity, GrayLevelVariance, HighGrayLevelEmphasis, LargeDependenceEmphasis, LargeDependenceHighGrayLevelEmphasis, LargeDependenceLowGrayLevelEmphasis, LowGrayLevelEmphasis, SmallDependenceEmphasis, SmallDependenceHighGrayLevelEmphasis, SmallDependenceLowGrayLevelEmphasis | 14 |
| GLRLM | GrayLevelNonUniformity, GrayLevelNonUniformityNormalized, GrayLevelVariance, HighGrayLevelRunEmphasis, LongRunEmphasis, LongRunHighGrayLevelEmphasis, LongRunLowGrayLevelEmphasis, LowGrayLevelRunEmphasis, RunEntropy, RunLengthNonUniformity, RunLengthNonUniformityNormalized, RunPercentage, RunVariance, ShortRunEmphasis, ShortRunHighGrayLevelEmphasis, ShortRunLowGrayLevelEmphasis | 16 |
| GLSZM | GrayLevelNonUniformity, GrayLevelNonUniformityNormalized, GrayLevelVariance, HighGrayLevelZoneEmphasis, LargeAreaEmphasis, LargeAreaHighGrayLevelEmphasis, LargeAreaLowGrayLevelEmphasis, LowGrayLevelZoneEmphasis, SizeZoneNonUniformity, SizeZoneNonUniformityNormalized, SmallAreaEmphasis, SmallAreaHighGrayLevelEmphasis, SmallAreaLowGrayLevelEmphasis, ZoneEntropy, ZonePercentage, ZoneVariance | 16 |
| NGTDM | Busyness, Coarseness, Complexity, Contrast, Strength | 5 |

Note: GLCM, grey level co-occurrence matrix (GLCM) features. GLDM, gray level dependence matrix. GLRLM, grey-level run length matrix. GLSZM, grey-level size zone matrix.NGTDM, neighbouring gray tone difference matrix
