# Supplementary material for: Multimodal striatal neuromarkers in distinguishing parkinsonian variant of multiple system atrophy from idiopathic Parkinson's disease
Source: CNS Neurosci Ther. 2022 Sep 1;28(12):2172–82. doi: 10.1111/cns.13959 (PMC9627351; doi:10.1111/cns.13959)
Supplement: Supplementary file 6 — Appendix S6 [file CNS-28-2172-s004.docx]

**The selected features in each model**

**“Function” model**

Step 1: mRMR (32):

mALFF: left dorsolateral putamen

mALFF: left dorsal caudate

mReHo: left dorsolateral putamen

mReHo: left dorsal caudate

outFC: left dorsolateral putamen-right A4upper limber region of precentral gyrus

outFC: left dorsolateral putamen-right A6medial area of superior frontal gyrus

outFC: left dorsolateral putamen-left A14medial area of orbital gyrus

outFC: left dorsolateral putamen-inferior frontal sulcus of inferior frontal gyrus

outFC: left dorsolateral putamen-A8ventrolateral area of middle frontal gyrus

outFC: left dorsolateral putamen-right pre-motor thalamus

outFC: left dorsolateral putamen-left lateral pre-frontal thalamus

outFC: left dorsolateral putamen- A4hf area of precentral gyrus

inFC: left dorsal caudate-right nucleus accumbens

outFC: left dorsal caudate-right A9lateral area of superior frontal gyrus

outFC: left dorsal caudate-right A9/46dorsal area of middle frontal gyrus

outFC: left dorsal caudate-right A38medial area of superior temporal gyrus

outFC: left dorsal caudate-right A45caudal area of inferior frontal gyrus

outFC: right dorsal caudate- right A45caudal area of inferior frontal gyrus

outFC: right dorsal caudate-left A21rostral area of middle temporal gyrus

outFC: right dorsal caudate-right A10medial area of superior frontal gyrus

inFC: right dorsal caudate -right dorsolateral putamen

outFC: right dorsolateral putamen-left A6 medial area of superior frontal gyrus

inFC: right dorsolateral putamen-right dorsal caudate

outFC: right dorsolateral putamen-right A6medial area of superior frontal gyrus

outFC: right dorsolateral putamen-left A4hf area of precentral gyrus

outFC: right ventromedial putamen-left A10lateral area of middle frontal gyrus

outFC: left dorsolateral putamen-right cerebellum IV

outFC: left dorsal caudate -right cerebellum IV

outFC: left dorsolateral putamen-right cerebellum V

outFC: left dorsolateral putamen-right cerebellum VI

outFC: right dorsal caudate-left cerebellum II

outFC: left dorsal caudate-left cerebellum II

Step 2: LASSO (12):

mALFF: left dorsolateral putamen

outFC: left dorsolateral putamen-right A4upper limber region of precentral gyrus

outFC: left dorsolateral putamen-right cerebellum IV

inFC: left dorsal caudate-right nucleus accumbens

outFC: left dorsal caudate-right A9lateral region of superior frontal gyrus

outFC: left dorsolateral putamen-right A6medial area of superior frontal gyrus

outFC: left dorsal caudate -right cerebellum IV

outFC: left dorsolateral putamen-right cerebellum V

outFC: left dorsal caudate-right A9/46dorsal area of middle frontal gyrus

outFC: right ventromedial putamen-left A10lateral area of middle frontal gyrus

outFC: right dorsolateral putamen-left A6medial area of superior frontal gyrus

outFC: left dorsolateral putamen-left lateral pre-frontal thalamus

**“Diffusion” model**

Step 1: mRMR (6):

FA: left dorsolateral putamen

MD: left dorsolateral putamen

FA: left dorsal caudate

MD: right dorsolateral putamen

FA: right dorsolateral putamen

MD: left ventromedial putamen

Step 2: LASSO (2):

MD: left dorsolateral putamen

FA: left dorsal caudate

**“Volumetry” model**

Step 1: mRMR (5):

Volume: left dorsolateral putamen

Volume: left dorsal caudate

Volume: right dorsolateral putamen

Volume: right dorsal caudate

Volume: left ventromedial putamen

Step 2: LASSO (2):

Volume: left dorsolateral putamen

Volume: right dorsolateral putamen

**“Iron” model**

Step 1: mRMR (8):

Iron: Entropy: left dorsolateral putamen

Iron: Variance: left dorsolateral putamen

Iron: GLCM-DifferenceEntropy: left dorsolateral putamen

Iron: GLCM-SumEntropy: left dorsolateral putamen

Iron: GLRLM-RunLengthNonUniformityNormalized: left dorsolateral putamen

Iron: GLSZM-SizeZoneNonUniformityNormalized: left dorsolateral putamen

Iron: GLSZM- GrayLevelVariance: left dorsal caudate

Iron: GLSZM- GrayLevelNonUniformity: left dorsolateral putamen

Step 2: LASSO (4):

Iron: Entropy: left dorsolateral putamen

Iron: GLCM-DifferenceEntropy: left dorsolateral putamen

Iron: GLRLM-RunLengthNonUniformityNormalized: left dorsolateral putam

ron: GLSZM-SizeZoneNonUniformityNormalized: left dorsolateral putamen

**“Multimodal” model**

Step 1: mRMR (55):

outFC: left dorsolateral putamen-right A4upper limber region of precentral gyrus

outFC: left dorsolateral putamen-right A6medial area of superior frontal gyrus

outFC: left dorsolateral putamen-left A14medial area of orbital gyrus

outFC: left dorsolateral putamen-inferior frontal sulcus of inferior frontal gyrus

outFC: left dorsolateral putamen-A8ventrolateral area of middle frontal gyrus

outFC: left dorsolateral putamen-right pre-motor thalamus

outFC: left dorsolateral putamen-left lateral pre-frontal thalamus

outFC: left dorsolateral putamen- A4hf area of precentral gyrus

outFC: left dorsolateral putamen-right A4t area of precentral gyrus

outFC: left dorsal caudate-right A9lateral area of superior frontal gyrus

outFC: left dorsal caudate-right A9/46dorsal area of middle frontal gyrus

outFC: left dorsal caudate-right A38medial area of superior temporal gyrus

outFC: left dorsal caudate-right A45caudal area of inferior frontal gyrus

outFC: left dorsal caudate-left A22 rostral area of middle temporal gyrus

outFC: right dorsal caudate- right A45caudal area of inferior frontal gyrus

outFC: right dorsal caudate-left A21rostral area of middle temporal gyrus

outFC: right dorsal caudate-right A10medial area of superior frontal gyrus

outFC: right dorsal caudate-left A45rostral area of inferior frontal gyrus

outFC: right dorsolateral putamen-left A6medial area of superior frontal gyrus

outFC: right dorsolateral putamen-right A6medial area of superior frontal gyrus

outFC: right dorsolateral putamen-left A4hf area of precentral gyrus

outFC: right ventromedial putamen-left A10lateral area of middle frontal gyrus

outFC: left ventromedial putamen- left A22 rostral area of middle temporal gyrus

outFC: right ventromedial putamen-right A10lateral area of middle frontal gyrus

outFC: right ventromedial putamen-left A38medial area of superior temporal gyrus

outFC: left dorsolateral putamen-right cerebellum IV

outFC: left dorsal caudate -right cerebellum IV

outFC: left dorsolateral putamen-right cerebellum V

outFC: left dorsolateral putamen-right cerebellum VI

outFC: right dorsal caudate-left cerebellum II

outFC: left dorsal caudate-left cerebellum II

inFC: left dorsal caudate-right nucleus accumbens

inFC: right dorsal caudate -right dorsolateral putamen

inFC: right dorsolateral putamen-right dorsal caudate

mALFF: left dorsolateral putamen

mALFF: left dorsal caudate

mALFF: right dorsolateral putamen

mALFF: right dorsal caudate

mReHo: left dorsolateral putamen

mReHo: left dorsal caudate

FA: left dorsolateral putamen

MD: left dorsolateral putamen

FA: left dorsal caudate

MD: right dorsolateral putamen

Volume: left dorsolateral putamen

Volume: left dorsal caudate

Volume: right dorsolateral putamen

Iron: Entropy: left dorsolateral putamen

Iron: Variance: left dorsolateral putamen

Iron: GLCM-DifferenceEntropy: left dorsolateral putamen

Iron: GLCM-SumEntropy: left dorsolateral putamen

Iron: GLRLM-RunLengthNonUniformityNormalized: left dorsolateral putamen

Iron: GLSZM-SizeZoneNonUniformityNormalized: left dorsolateral putamen

Iron:GLSZM- GrayLevelVariance: left dorsal caudate

Iron: GLSZM- GrayLevelNonUniformity: left dorsolateral putamen

Step 2: LASSO (16):

mALFF: left dorsolateral putamen

outFC: left dorsolateral putamen-right A4upper limber region of precentral gyrus

inFC: left dorsal caudate-right nucleus accumbens

outFC: left dorsolateral putamen-right A6medial area of superior frontal gyrus

outFC: left dorsal caudate-right A9lateral area of superior frontal gyrus

outFC: left dorsal caudate-right A9/46dorsal area of middle frontal gyrus

outFC: right dorsolateral putamen-left A6 medial area of superior frontal gyrus

outFC: right ventromedial putamen-left A10lateral area of middle frontal gyrus

outFC: left dorsolateral putamen-right cerebellum IV

outFC: left dorsal caudate -right cerebellum IV

outFC: left dorsolateral putamen-right cerebellum V

MD: left dorsolateral putamen

Volume: left dorsolateral putamen

Iron Entropy: left dorsolateral putamen

Iron: GLCM DifferenceEntropy: left dorsolateral putamen

Iron GLRLM- RunLengthNonUniformityNormalized: left dorsolateral putamen
