# Supplementary material for: Multimodal striatal neuromarkers in distinguishing parkinsonian variant of multiple system atrophy from idiopathic Parkinson's disease
Source: CNS Neurosci Ther. 2022 Sep 1;28(12):2172–82. doi: 10.1111/cns.13959 (PMC9627351; doi:10.1111/cns.13959)
Supplement: Supplementary file 7 — Appendix S7 [file CNS-28-2172-s007.docx]

The Performance of “Multimodal” and “Clinical-Multimodal” models in the subgroup

| Model | AUC | B-ACC | Sensitivity | Specificity | PPV | NPV |
| --- | --- | --- | --- | --- | --- | --- |
| Early-stage subgroup |  |  |  |  |  |  |
| Multimodal | 0.877  (0.637,0.982) | 0.899^*^ | 0.889 | 0.889 | 0.889 | 0.889 |
| Clinial-Multimodal | 0.951  (0.736,0.999) | 0.899^*^ | 0.889 | 0.889 | 0.889 | 0.889 |
| Moderately advanced stage subgroup |  |  |  |  |  |  |
| Multimodal | 0.967  (0.817,0.999) | 0.926^*^ | 0.923 | 0.929 | 0.923 | 0.929 |
| Clinial-Multimodal | 0.973  (0.825,0.999) | 0.921^*^ | 0.923 | 0.929 | 0.923 | 0.929 |

AUC: area under the receiving operator curve; B-ACC: balanced accuracy; PPV: positive predictive value; NPV: negative predictive value. * denotes *p*<0.001 under permutation test (1,000 times).
