# Supplementary material for: Multimodal striatal neuromarkers in distinguishing parkinsonian variant of multiple system atrophy from idiopathic Parkinson's disease
Source: CNS Neurosci Ther. 2022 Sep 1;28(12):2172–82. doi: 10.1111/cns.13959 (PMC9627351; doi:10.1111/cns.13959)
Supplement: Supplementary file 9 — Appendix S9 [file CNS-28-2172-s003.docx]

The optimized hyperparameters of the models

| Model | Lambda | C | Gamma | |
| --- | --- | --- | --- | --- |
| Function | 0.035 | 0.680 | | 0.005 |
| Diffusion | 0.028 | 1.260 | | 0.020 |
| Volumetry | 0.022 | 1.714 | | 0.020 |
| Iron | 0.933 | 0.031 | | 0.020 |
| Multimodal | 0.019 | 0.926 | | 0.003 |
| Clinical-multimodal | 0.019 | 0.926 | | 0.003 |
